# The Relationship between Nrf2 and HO-1 with the Severity of COVID-19 Disease

**DOI:** 10.3390/medicina58111658

**Published:** 2022-11-16

**Authors:** Damir Mihić, Domagoj Loinjak, Lana Maričić, Robert Smolić, Ines Šahinović, Kristina Steiner, Sven Viland, Vatroslav Šerić, Mario Duvnjak

**Affiliations:** 1 Faculty of Medicine, J. J. Strossmayer University in Osijek, 31000 Osijek, Croatia; 2 Department of Pulmology and Intensive Care Medicine, University Center Hospital Osijek, 31000 Osijek, Croatia; 3 Department of Heart and Vascular Diseases, University Center Hospital Osijek, 31000 Osijek, Croatia; 4 Faculty of Dental Medicine and Health Osijek, J. J. Strossmayer University in Osijek, 31000 Osijek, Croatia; 5 Department of Clinical Laboratory Diagnostics, University Center Hospital Osijek, 31000 Osijek, Croatia; 6 Department of Endocrinology, University Center Hospital Osijek, 31000 Osijek, Croatia; 7 Department of Infective Diseases, University Center Hospital Osijek, 31000 Osijek, Croatia

**Keywords:** NF-E2-related factor 2 (Nrf2), heme oxygenase-1 (HO-1), COVID-19

## Abstract

Nuclear factor erythroid 2-related factor 2 (Nrf2) and heme oxygenase-1 (HO-1) have significant roles in the development of a hyperinflammatory state in infectious diseases. We aimed to investigate the association of the serum concentrations of Nrf2 and HO-1 with the severity of COVID-19 disease. The study included 40 subjects with mild and moderately severe forms of the disease (MEWS scoring system ≤2). Twenty of the subjects had MEWS scores of 3 or 4, which indicate a severe form of the disease, and twenty subjects had a MEWS score of ≥5, which indicates a critical form of the disease. HO-1 and Nrf2 were measured using the commercially available Enzyme-Linked Immunosorbent Assay (ELISA). Subjects with the most severe form of COVID-19 (critically ill) had a lower concentration of Nrf2 that negatively correlated with the markers of hyperinflammatory response (CRP, IL-6, ferritin). This observation was not made for HO-1, and the correlation between Nrf2 and HO-1 values was not established. In the mild/moderate form of COVID-19 disease, Nrf2 was associated with an increased 1,25 dihydroxy vitamin D concentration. The results of this study show that Nrf2 has a role in the body’s anti-inflammatory response to COVID-19 disease, which makes it a potential therapeutic target.

## 1. Introduction

COVID-19 (coronavirus disease 2019) is caused by the new SARS-CoV-2 virus, which was first isolated in China in December 2019. The virus has since spread to all parts of the world, causing a pandemic that became a major global public health and clinical problem [1,2].

The spectrum of clinical manifestations of COVID-19 disease is broad, from asymptomatic, mild and moderately severe forms that manifest as a cold and a flu-like illness, to severe forms that manifest as severe pneumonia and acute respiratory distress syndrome [3,4]. Despite numerous studies, it is still not known why some individuals are prone to developing more severe forms of the disease than others. Studies have shown that age, diabetes, obesity and arterial hypertension are some of the most frequent risk factors in the development of more severe forms of the disease [5]. 

One of the main characteristics of severe and critical manifestations of COVID-19 disease is the hyperinflammatory response of the immune system, which is characterized by the increased production of proinflammatory cytokines. This increased production is directly related to the development of acute respiratory distress syndrome and multiple organ failure syndrome [6,7,8]. 

Besides the hyperinflammatory state, the development of oxidative stress is also recognized as an important pathophysiological mechanism in the development of severe and critical forms of COVID-19 disease [9,10]. Mitochondrial dysfunction caused by the penetration of the virus into cells is considered the main source of the increased concentration of reactive oxygen species (ROS). Hyperinflammation itself contributes to oxidative stress by stimulating the Fenton reaction (Fe^2+^ + H_2_O_2_ → Fe^3+^ + HO^−^ + HO^•^) via proinflammatory mediators, especially interleukin-6 (IL-6) and ferritin [11,12]. 

One of the most explored responses of the organism to oxidative stress is based on the transcription factor, nuclear factor and erythroid 2-related factor (Nrf2). Under normal circumstances, Nrf2 can be found in the cytoplasm of the cell bound to its inhibitor Kelch-like ECH-associated protein 1 (Keap 1) [13]. In the presence of ROS, the Keap 1–Nrf2 complex dissociates and Nrf2 migrates from the cytoplasm to the nucleus, where it stimulates the transcription of genes whose products participate in the antioxidant response (ARE, antioxidant response element). ARE products are primarily enzymes with the ability to inactivate ROS [14]. The role of Nrf2 expression in viral infections of the respiratory tract (respiratory syncytial virus, type A influenza virus) was studied in animal models. Nrf2 showed to have a protective role in the development of oxidative stress that causes more severe forms of viral respiratory diseases [15,16]. In addition, research has shown that Nrf2 activation, besides its antioxidant effect, is also associated with an anti-inflammatory effect, which is reflected in the reduced expression and secretion of inflammatory proteins such as proinflammatory cytokines [17,18]. Nrf2 also showed an antiviral effect, including the suppression of virus replication, which was demonstrated in cell cultures infected with SARS-CoV-2 virus, Zika virus and Herpes simplex virus [19]. One of the significant exogenous inducers of Nrf2 activity is 1,25 dihydroxy vitamin D, which explains its antioxidant effect and the possible effect on the course and severity of COVID-19 disease [20,21]. 

One of the ARE enzymes is heme oxygenase-1 (HO-1), which has been proven to have anti-inflammatory, antioxidant and apoptotic effects [22]. HO-1 antioxidant effect is based on the catabolism of the prooxidative heme to its basic units: iron, carbon monoxide and biliverdin, which soon turns into bilirubin [23]. The increase in HO-1 activity during the acute inflammatory response is involved in the tissue protection from oxidative stress caused by ROS and the suppression of the inflammatory response [24]. 

Based on the described characteristics of Nrf2 and HO-1, they are interesting in terms of examining their roles in the mechanisms of the development of more severe forms of COVID-19 disease, as well as possible therapeutic targets in disease treatment [25,26,27,28,29,30,31,32]. Despite this, there are few clinical studies that have investigated the value of Nrf2 and HO-1 in humans with COVID-19, such as Detsik et al.’s study, which found lower Nrf2 values in symptomatic compared to asymptomatic pediatric patients [33,34]. 

The main aim of this study was to examine the association of Nrf2 and HO-1 in COVID-19 patients with the disease severity and the markers of the hyperinflammatory response (C-reactive protein-CRP, IL-6, ferritin). A secondary objective was to examine the influence of demographic characteristics of the subjects (age, gender), comorbidities and serum concentrations of 1,25 dihydroxy vitamin D on the values of Nrf2 and HO-1 in patients with COVID-19 disease.

## 2. Materials and Methods

A cross-sectional study was conducted during the second and the third epidemic wave of COVID-19 disease in the Republic of Croatia. The study was approved by the Ethics Committee of the Faculty of Medicine, Josip Juraj Strossmayer University in Osijek (approval number 2158-61-07-21-189). In total, 80 adult patients were included with an average age of 60 (27–79), equally represented by gender (51% women and 49% men). All subjects had a symptomatic form of the disease and were treated at the Clinical Hospital Centre Osijek, Croatia. COVID-19 was confirmed by a PCR test. The basis of this study was: (1) the collection of the subject’s demographic and clinical data; (2) the assessment of the severity of the disease at the time of peripheral venous blood sampling, based on the Modified Early Warning scoring system (MEWS); and (3) peripheral venous blood sampling to determine the serum concentrations of Nrf2 and HO-1, the markers of the hyperinflammatory response (CRP, IL-6, ferritin) and 1,25 dihydroxy vitamin D values. The MEWS scoring system is based on scoring the values of individual vital parameters (respiratory rate, heart rate, systolic blood pressure, body temperature, state of consciousness), where a score of ≤2 indicates a mild or a moderately severe form of the disease, 3–4 a severe form of the disease and ≥5 a critical form of the disease.

Based on the MEWS scoring system, the subjects were divided into three groups: 40 subjects with mild and moderately severe forms of COVID-19 disease (MEWS score of ≤2), 20 subjects with a severe form of COVID-19 disease (MEWS score of 3 or 4) and 20 subjects with a critical form of COVID-19 disease (MEWS score of ≥5). The study did not include patients with an asymptomatic form of COVID-19 disease, patients affected with diseases in which an association with reduced Nrf2 values was previously established (autoimmune connective tissue diseases, neurodegenerative diseases), patients with chronic corticosteroid and/or immunomodulatory therapy and patients who were in an active oncology treatment (chemotherapy, immunotherapy).

Data: The data on the subjects were documented from electronic medical history, which included basic demographic data (gender, age) and present comorbidities. From the subjects with mild and moderately severe forms of COVID-19 disease, medical data were also collected through interviews. The severity of the disease was assessed at the time of blood sampling. 

Samples: Blood samples were obtained by venipuncture and collected in two 4 mL serum-separating tubes. The samples were centrifuged at 1370 g for 10 min. CRP, IL-6 and ferritin were determined in the first blood sample immediately after sample centrifugation. From the second blood sample, serum aliquots were separated immediately after sampling and frozen at −70 °C until the analysis of 1,25 dihydroxy vitamin D, HO-1 and Nrf2. 

CRP and ferritin were measured on the biochemistry analyzer Olympus AU680 (Beckman Coulter, Brea, CA, USA) using a latex-enhanced immunoassay method. 1,25 dihydroxy vitamin D was determined using liquid chromatography coupled to mass spectrometry (LC-MS/MS) on LCMS-8060 (Shimadzu Corporation, Kyoto, Japan), with a commercially available method from Chromsystems (Gräfelfing, Germany). Serum IL-6, HO-1 and Nrf2 were measured using an Enzyme-Linked Immunosorbent Assay (ELISA). IL-6 and HO-1 levels were measured using a sandwich ELISA kit for IL-6 (Invitrogen, Thermo Fisher Scientific, Waltham, MA, USA) and HO-1 (Abcam, Cambridge, UK), respectively. Nrf2 was measured using a competitive ELISA kit (MyBioSource, San Diego, CA, USA). ELISA tests were performed according to the manufacturer’s instructions on the ELISA processor EtiMax 3000 (DiaSorin, Saluggia, Italy). The HO-1 and Nrf2 intra-assay performance characteristics were ≤3.1% and ≤4.2%, respectively.

Based on the previous studies focusing on Nrf2 and HO-1, the sample size was estimated a priori using G-power 3.1. The power analyses revealed that 80 participants would give 85% power at alpha = 0.05 to detect medium effect size using F tests. Skewness values were determined to be outside normality at the *p* < 0.05 significance level if the value was greater than the absolute value of 3; kurtosis values were outside of normality when the absolute value reached 8 or 10. Therefore, the skewness and kurtosis of the variables in this study were within the ±2 absolute value range, indicating that they did not deviate from the assumption of a normal distribution. Given this, the groups were compared using either the *t*-test (e.g., gender differences) or ANOVA (e.g., groups with a different severity of COVID-19), whereas the relationships between the variables of interest were examined using the Pearson correlation coefficients. The results are expressed as mean and standard deviation. The statistical significance was set at *p* < 0.05, and at *p* < 0.01for the correlation analysis. The statistical analysis was carried out using IBM SPSS Statistics 20; Chicago, IL, USA).

## 3. Results

Basic demographic and clinical data according to COVID-19 disease severity are presented in Table 1.

**Figure 1:**
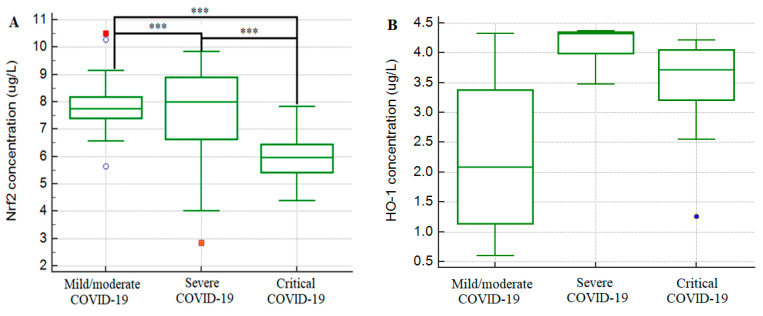
Serum concentrations of Nrf2 (**A**) and HO-1 (**B**) in patients with COVID-19 disease. *** *p* < 0.05.

The average value of Nrf2 among all subjects was 6.70 ± 1.65 μg/L, while the average value of HO-1 was 3.56 ± 0.76 μg/L. Figure 1 shows the values of Nrf2 and HO-1 according to the groups of subjects (mild/moderate, severe and critical). The correlation between Nrf2 and HO-1 was low and not statistically significant (r = 0.04, *p* = 0.724).

The correlation of serum concentrations of Nrf2 and HO-1 with the severity of the disease is shown in Table 2. A statistically significant difference in concentrations of Nrf2 was recorded in all three groups of subjects, with the highest concentration found in the group with a severe form of the disease (8.15 ± 1.08 μg/L), followed by subjects with a mild and a moderate form of the disease (6.67 ± 1.58 μg/L), and the lowest concentration of Nrf2 was found in patients with a critical form of the disease (5.30 ± 0.86 μg/L) (*p* < 0.001). Regarding HO-1 concentration, the Bonferroni post hoc test revealed a statistically significant difference only between the group with a mild and moderate form of the disease and the group with a severe form of the disease (*p* = 0.049), whereby patients with a severe form of the disease had a significantly higher concentration of HO-1.

The days of illness at sampling ranged from 3 to 23 days (12.08 ± 4.72). When it comes to the relationship between the concentrations of Nrf2 and HO-1 and the day of sampling, no significant correlations were found. Thus, the correlation of Nrf2 concentration with the sampling day was r = 0.04, *p* = 0.710, and the correlation of HO-1 was r = 0.11, *p* = 0.325. The concentrations of these two factors were equal regardless of the day of the disease at sampling.

As the severity of COVID-19 disease is directly related to the enhanced immune response that causes the cytokine storm, we examined the correlation of CRP, IL-6 and ferritin with the severity of the disease and the correlation between Nrf2 and HO-1 and hyperinflammatory response biomarkers (CRP, IL-6 and ferritin), presented in Table 3 and Table 4.

We observed a significant negative correlation between Nrf2 and proinflammatory biomarkers (CRP, IL-6 and ferritin). This association was the strongest in the group of critically ill subjects, and moderately strong in the group of severe COVID-19 disease. In the mild and moderate group of COVID-19 disease, the correlation of Nrf2 and proinflammatory biomarkers was not observed.

The serum values of HO-1 among all subjects were not significantly associated with any of the hyperinflammatory response biomarkers. In the group of subjects with a mild and moderately severe form of the disease, a significant negative, albeit low, correlation was found between the values of HO-1 and IL-6 and ferritin. Similarly, in the group of patients with a severe form of the disease, a higher concentration of HO-1 was associated with lower concentrations of ferritin. In the group of patients with a critical form of the disease, no statistically significant correlation of HO-1 concentration with any marker of hyperinflammatory response was found.

HO-1 showed to be significantly lower in females compared to males. For Nrf2 concentration, we found no difference in the serum concentration of Nrf2 between genders (Table 5). The correlation analysis was used to examine the relationship between the age and serum concentrations of Nrf2 and HO-1, and there was no significant correlation (r = −0.04, *p* = 0.761 and r = −0.02, *p* = 0.864, respectively).

The influence of comorbidities on the serum concentrations of Nrf2 and HO-1 was tested using the correlation analysis, as shown in Table 6.

We found a weak positive correlation of Nrf2 with obesity, except in the group with mild and moderate COVID-19, where Nrf2 negatively correlated with obesity. A weak negative correlation of Nrf2 was observed in subjects with diabetes mellitus. In contrast, serum HO-1 concentrations were not significantly associated with any of the comorbidities.

Negative correlations of Nrf2 concentration with obesity, hyperlipidemia and chronic obstructive pulmonary disease were found in the group of patients with mild and moderately severe clinical presentations of the disease, as well as a negative correlation with diabetes in the group of subjects with a severe form of the disease. In the group of patients with a critical form of the disease, no significant correlation was found between the Nrf2 concentrations and comorbidities. Serum HO-1 concentrations were not significantly associated with any of the comorbidities.

When comparing the MEWS groups, the subject with a mild or moderate form of the disease had a significantly higher concentration of 1,25 dihydroxy vitamin D than the subjects with a critical form (*p* < 0.001) and the subjects with a severe form of the disease (*p* < 0.01). However, subjects with a severe and critical form of the disease did not differ significantly from each other (Table 7).

The correlation analysis was used to examine the relationship between the serum concentration of Nrf2 and HO-1 and the concentration of 1,25 dihydroxy vitamin D (Table 8). A higher Nrf2 concentration was correlated with a higher concentration of 1,25 dihydroxy vitamin D in all subjects and subjects with a mild/moderate COVID-19 disease, but the same correlations were not observed for the HO-1 concentration.

## 4. Discussion

In this study, we aimed to determine the association of serum concentrations of Nrf2 and HO-1 with the severity of COVID-19 disease. The results of this study showed that patients with the most severe forms of COVID-19 disease (critically ill) had lower values of Nrf2 and that it was negatively correlated with the markers of hyperinflammatory response (CRP, IL-6, ferritin). HO-1 showed no significant difference according to COVID-19 severity, nor a correlation with Nrf2 values. This may be due to the influence of other pathways on HO-1 expression, such as mitogen-activated protein kinases (MAPK) and phosphatidylinositol-3 kinase (PI3K) [35].

The results of our study suggest an anti-inflammatory effect of the nuclear factor Nrf2 on COVID-19 disease, which is what other studies also showed. To the best of our knowledge, this is the first study to report a high negative correlation of Nrf2 and proinflammatory biomarkers CRP, IL-6 and ferritin in humans with the critical form of COVID-19 disease. The activation of Nrf2 results in the suppression of the immune response not only by enhancing the antioxidant response of the cell, but also by inhibiting the secretion of proinflammatory cytokines in human macrophages such as IL-6 and TNFα [36,37]. It has also been described that the use of Nrf2 inducers can reduce the inflammatory response in patients with acute and chronic inflammatory diseases [38]. Given that the severity of COVID-19 disease is related to the strength of the virus-induced oxidative stress, as well as the development of a hyperinflammatory response and a cytokine storm, it is to be expected that the concentration of Nrf2 depends on the severity of the disease and that it can influence the severity of the disease [39]. In this study, patients who had a mild or moderate and a severe form of the disease according to the MEWS scoring system had significantly higher levels of NrF2 and lower values of hyperinflammatory response markers, in contrast to patients with a critical form of the disease, who had the lowest levels of Nrf2 and the highest levels of hyperinflammatory markers. The patients with a mild and moderate form of the disease had slightly lower Nrf2 values compared to the patients with a severe form of the disease, which can be explained by a reduced viral induction of oxidative stress and the associated activation and the release of Nrf2. It is important to emphasize that patients with a severe form were treated with oxygen (nasal cannula or high flow oxygen therapy), and oxygen therapy itself creates additional oxidative stress that induces Nrf2 [40].

Given that the activation of Nrf2 results in antiviral activity, some authors also assume that its lower values in viral infections may also be the result of a direct viral effect on its reduced expression to ensure a smoother replication [19].

With this explanation, Gumus et al. justifies the lower values of Nrf2 in symptomatic compared to asymptomatic COVID-19 pediatric patients [34], and Olagnier et al. uses this mechanism to describe a decrease in the Nrf2 expression in the lung biopsies of patients suffering from ARDS caused by the SARS-CoV2 virus. Olagnier et al. also proved that Nrf2 activators can inhibit the replication of the SARS-CoV2 virus itself [41], which helps to understand the connection between the reduced Nrf2 values and the progression to more severe forms of COVID-19 disease.

Lower Nrf2 values in critically ill subjects can also be affected by chronic comorbidities. We found significantly lower concentrations of Nrf2 in obese patients and patients with diabetes mellitus, and these are proven to be one of the most significant risk factors in the development of severe forms of COVID-19 disease [5]. Lower values of Nrf2 in patients with diabetes mellitus, as well as the positive effects of using Nrf2 inducers in these patients, were described by various authors [42,43,44]. Moreover, the dysregulation of Nrf2 and its reduced concentration have been described in obesity and might contribute to more severe inflammation and related disease complications seen in obese people [45]. In this study, obesity and diabetes mellitus were most prevalent in patients with a critical form of COVID-19 disease—50% of critically ill patients had one or both comorbidities, which could contribute to the reduced Nrf2 values.

Following previous studies that placed 1,25 dihydroxy vitamin D in the context of the Nrf2 activator [46,47], this research showed a positive correlation between the values of 1,25 dihydroxy vitamin D and Nrf2 in the mild/moderate form of the disease, and its possible positive role in the treatment and prevention of COVID-19 [48]. However, when looking at the severe and critically ill patients, this relationship was not so strong, indicating different or even blocked effects of 1,25 dihydroxy vitamin D in severe and critical forms of COVID-19 disease. The suboptimal status of 1,25 dihydroxy vitamin D in the group of patients with a critical form of the disease is in accordance with the current knowledge because it is precisely the low level of 1,25 dihydroxy vitamin D that is responsible for the reduced expression of endogenous antimicrobial peptides and ineffective regulation of innate as well as acquired immunity. Consequently, as expected in the group with a critical form of the disease, lower Nrf2 values are correlated with lower 1,25 dihydroxy vitamin D values.

Lower values of Nrf2 in critically ill subjects can also be a result of different external and endogenous factors, such as polymorphisms and somatic mutations of the Nrf2 gene, that could result in a lower expression and Nrf2 concentration [49,50]. Because the research of Nrf2 gene polymorphisms and mutation was not in the scope of this study, we cannot tell to what extent it contributed to our study’s results.

Although the serum values of HO-1 within all subjects were not significantly related to any markers of the hyperinflammatory response, certain correlations were found within groups, although they are not as strong as Nrf2 correlations. In the group of mild and moderate forms of the disease, a higher concentration of HO-1 was correlated with lower concentrations of ferritin and IL-6, while in the group of severely ill patients, HO-1 was correlated only with a lower ferritin concentration. This effect of an anti-inflammatory role of HO-1 was previously described by Maines [51].

Regarding the relationship between the severity of the disease and the values of HO-1, we found that all patients with a severe form of the disease had higher values of HO-1 concentration compared to those with the mild and moderate form and the critical form of COVID-19 disease. This differs from the study of Detsik et al., who examined the expression of HO-1 and found that the serum concentration of HO-1 mRNA was higher in the group of critically ill COVID-19 patients compared to the group of severely ill patients [33]. These differences could have been influenced by the time of blood sampling to analysis and the duration of the disease before blood sampling. In our study, the duration of the disease on the blood sampling day was longer for critically ill patients (15.30 ± 2.66 days) compared to the previously mentioned study (5.77 ± 2.98 days). Additionally, the pO_2_/FiO_2_ ratio, which reflects the severity of pneumonia and the presence of ARDS, was lower in our study (76.90 ± 23.16 vs. 137.90 ± 79.23, respectively). Considering the small sample groups (both studies included 20 critically ill patients), it is necessary to conduct studies with a larger number of subjects and a serial monitoring of HO-1 values.

Furthermore, unlike Nrf2, serum values of HO-1 were not significantly correlated with any of the comorbidities, with a few exceptions in some groups of patients. A low correlation was found between HO-1 in patients and arterial hypertension in the group of severely ill patients and in patients with diabetes mellitus and coronary disease in the group of critically ill patients. Although by reviewing the literature, we can find studies that discuss the protective effect of HO-1 in treating arterial hypertension [52], there are no studies in humans with arterial hypertension. Some studies indicate that the serum values of HO-1 are reduced in patients with diabetes mellitus [53,54].

Although 1,25 dihydroxy vitamin D is described as an inducer of the Nrf2/HO-1 pathway in the defense against oxidative stress [55], in our study, its values were positively correlated only with Nrf2; we did not observe a correlation with HO-1.

The limitation of this research is the small number of subjects within certain subgroups, especially the small number of respondents with a severe and critical form of COVID-19 disease, which is what we also found in other similar studies.

## 5. Conclusions

This study showed that patients with the most severe forms of COVID-19 disease had reduced Nrf2 values, together with increased values of hyperinflammatory response markers. Nrf2 showed a strong negative association with proinflammatory markers CRP, IL-6 and ferritin, especially in the critical, life-threatening form of COVID-19 disease. Anti-inflammatory effects of Nrf2 open the possibility of a potential therapeutic target in the treatment of severe COVID-19 disease, but further studies are required.

## Figures and Tables

**Table 1:** Patient demographic and clinical data according to COVID-19 disease severity.

	Mild/Moderate COVID-19 (*n* = 40)	Severe COVID-19 (*n* = 20)	Critical COVID-19 (*n* = 20)	Comparisons χ^2^/ANOVA	
**Demographic characteristics**					
Age (range)	53 (27–77)	66 (47–77)	67 (57–79)	15.79 ***	Mild/moderate < Severe ***; Mild/moderate < Critical ***
Male, (%)	40%	45%	70%	3.35	
**Clinical characteristics**					
Arterial hypertension, (%)	40%	55%	45%	1.21	
Diabetes mellitus, (%)	17%	39%	50%	10.16 **	
Obesity, (%)	7.5%	10%	35%	8.45 *	
Hyperlipidemia, (%)	5%	30%	30%	8.66 *	
Coronary artery disease, (%)	2.5%	15%	10%	3.24 a	
Days of illness before blood sampling ^1^	8.53 ± 3.42	15.95 ± 2.87	15.30 ± 2.66	52.17 ***	Mild/moderate < Severe ***; Mild/moderate < Critical ***
PaO_2_/FiO_2_ ratio *	383.85 ± 37.84	127.80 ± 43.01	76.90 ± 23.16	612.75 ***	Critical > Mild/moderate ***; Critical > Severe ***; Severe > Mild/moderate ***

χ^2^= chi-square test (categorical variables); ANOVA = analysis of variance (continuous variables). *** *p* < 0.001; ** *p* < 0.01; * *p* < 0.05; a = some cells have expected count less than 5. ^1^ Data are presented as mean ± standard deviation.

**Table 2:** Correlation of concentration of Nrf2 and HO-1 with severity of the disease.

	Mild/Moderate COVID-19 (*n* = 40)		Severe COVID-19 (*n* = 20)		Critical COVID-19 (*n* = 20)		ANOVA	Post Hoc Test
	*M*	*SD*	*M*	*SD*	*M*	*SD*	*F*-ratio	Bonferroni
**Nrf2 (** **μ** **g/L)**	6.67	1.58	8.15	1.08	5.30	0.86	23.33 **	Mild/moderate > Critical ** Severe > Mild/moderate ** Severe > Critical **
**HO-1 (** **μ** **g/L)**	3.45	0.88	3.95	0.22	3.45	0.75	3.38 *	Severe > Mild/moderate *

** *p* < 0.001; * *p* < 0.05; M = mean; SD = standard deviation.

**Table 3:** Correlation of proinflammatory biomarkers CRP, IL-6 and ferritin with the severity of disease.

	Mild/Moderate COVID-19 (*n* = 40)		Severe COVID-19 (*n* = 20)		Critical COVID-19 (*n* = 20)		ANOVA	Post Hoc Test
	*M*	*SD*	*M*	*SD*	*M*	*SD*	*F*-ratio	Bonferroni
**CRP** **(mg/L)**	45.65	43.90	144.10	46.11	252.97	90.28	83.42 ***	Critical > Mild/moderate *** Critical > Severe *** Severe > Mild/moderate ***
**IL-6** **(ng/L)**	22.96	38.07	118.30	83.62	605.52	249.37	131.18 ***	Critical > Mild/moderate *** Critical > Severe *** Severe > Mild/moderate **
**Ferritin** **(µg/L)**	261.39	163.73	1002.60	529.84	3675.35	1587.16	111.98 ***	Critical > Mild/moderate *** Critical > Severe *** Severe > Mild/moderate **

*** *p* < 0.001; ** *p* < 0.01; CRP = C-reactive protein; IL-6 = interleukin 6; M = mean; SD = standard deviation.

**Table 4:** Intercorrelations of Nrf2 and HO-1 with CRP, IL-6 and ferritin.

	All COVID-19 (*n* = 80)		Mild/Moderate COVID-19 (*n* = 40)		Severe COVID-19 (*n* = 20)		Critical COVID-19 (*n* = 20)	
	Nrf2	HO-1	Nrf2	HO-1	Nrf2	HO-1	Nrf2	HO-1
**HO-1**	0.04	—	−0.13	—	0.03	—	−0.30	—
**CRP (mg/L)**	−0.34 **	0.06	0.01	−0.12	−0.38	−0.16	−0.93 ***	0.31
**IL-6 (ng/L)**	−0.49 ***	−0.03	0.28	−0.34 *	−0.65 ***	−0.17	−0.94 ***	0.24
**Ferritin (ug/L)**	−0.48 ***	−0.02	0.02	−0.31 *	−0.67 **	**−0.40** ^**†**^	−0.86 ***	0.19

*** *p* < 0.001; ** *p* < 0.01; * *p* < 0.05; ^†^ *p* < 0.10; CRP = C-reactive protein; IL-6 = interleukin 6.

**Table 5:** Correlation between gender and concentration of Nrf2 and HO-1.

	Male (*n* = 39)		Female (*n* = 41)		
	*M*	*SD*	*M*	*SD*	*p*
**Nrf2 (µg/L)**	7.16	1.53	7.44	1.34	0.379
**HO-1 (µg/L)**	3.33	1.07	2.78	1.35	0.048 *

* *p* < 0.05; *M* = mean; *SD* = standard deviation.

**Table 6:** Intercorrelations of Nrf2 and HO-1 with comorbidities.

	All COVID-19 (*n* = 80)		Mild/Moderate COVID-19 (*n* = 40)		Severe COVID-19 (*n* = 20)		Critical COVD-19 (*n* = 20)	
	Nrf2	HO-1	Nrf2	HO-1	Nrf2	HO-1	Nrf2	HO-1
**HO-1**	0.04	—	−0.13	—	0.03	—	−0.30	—
**Obesity**	0.30 **	0.08	−0.35 *	0.15	−0.20	0.08	−0.01	0.12
**Arterial hypertension**	−0.02	0.05	−0.16	0.05	*0.32*	*−0.21*	*−0.32*	0.01
**Diabetes mellitus**	−0.24 *	0.04	−0.10	−0.05	*−0.25*	0.15	*−0.36*	0.20
**Hyperlipidemia**	−0.05	0.12	−0.37 *	−0.02	*0.22*	0.15	0.03	0.19
**Coronary artery disease**	0.02	0.17	0.07	0.13	−0.06	0.10	0.08	*0.25*
**Chronic cardiomyopathy**	−0.05	0.10	−0.17	0.16	0.04	0.08	−0.01	0.02
**Chronic kidney disease**	−0.05	0.12	−0.16	0.13	0.06	0.07	0.09	0.07
**Asthma**	0.10	−0.13	0.17	−0.13	−0.03	0.05	0.19	0.14
**COPD**	−0.14	−0.02	**−0.27** ^**†**^	0.03	**0.40** ^**†**^	0.05	0.02	−0.07

** *p* < 0.01; * *p* < 0.05; ^†^ *p* < 0.10; COPD = chronic obstructive pulmonary disease.

**Table 7:** Correlation of 1,25 dihydroxy vitamin D with severity of the disease.

	Mild/Moderate COVID-19 (*n* = 40)		Severe COVID-19 (*n* = 20)		Critical COVID-19 (*n* = 20)		ANOVA	Post Hoc Test
	*M*	*SD*	*M*	*SD*	*M*	*SD*	*F*-ratio	Bonferroni
**Vit D (µg/L)**	18.54	8.66	11.56	6.69	10.01	4.44	11.11 ***	Mild/moderate > Critical *** Mild/moderate > Severe **

*** *p* < 0.001; ** *p* < 0.01; Vit D = 1,25 dihydroxy vitamin D.

**Table 8:** Intercorrelations of Nrf2 and HO-1 with 1,25 dihydroxy vitamin D.

	All COVID-19 (*n* = 80)		Mild/Moderate COVID-19 (*n* = 40)		Severe COVID-19 (*n* = 20)		Critical COVID-19 (*n* = 20)	
	Nrf2	HO-1	Nrf2	HO-1	Nrf2	HO-1	Nrf2	HO-1
**HO-1**	0.04	—	−0.13	—	0.03	—	−0.30	—
**Vit D** **(µg/L)**	0.23 *	0.02	**0.29 ^†^**	0.12	0.31	0.18	−0.06	0.34

* *p* < 0.05; ^†^ *p* < 0.10; Vit D = 1,25 dihydroxy vitamin D.

## Data Availability

The datasets generated and/or analyzed during the current study are not publicly available due to the fact that individual privacy could be compromised, but are available from the corresponding author on reasonable request.
